# Neonatal ascites, leading to the diagnosis of congenital dengue with plasma leakage

**DOI:** 10.1002/ccr3.9493

**Published:** 2024-10-21

**Authors:** Sakviseth Bin, Sethikar Im

**Affiliations:** ^1^ Neonatal Intensive Care Unit Calmette Hospital Phnom Penh Cambodia; ^2^ University of Health Sciences Phnom Penh Cambodia

**Keywords:** ascites, dengue hemorrhagic fever, neonatal sepsis, neonate, vertical transmission

## Abstract

Although Cambodia is a dengue‐endemic country, mother‐to‐child transmission of dengue virus has yet to be documented. We report a rare case for congenital dengue diagnosed by RT‐PCR in a 4‐day‐old neonate with ascites. The neonate was initially treated for suspected perinatal infection. The management was supportive.

## 1 INTRODUCTION

Dengue has emerged as the most widespread mosquito‐borne viral disease in the world. Over two decades, the World Health Organization (WHO) records a significant 10‐fold increase from 505,430 (2000) to 5.2 million cases in 2019.^1^ In Cambodia as one of the dengue‐endemic country, the 19‐year national surveillance study recorded a total of 353,270 cases, with an average age‐adjusted incidence of 1.75 cases per 1000 persons per year and average annual fatality rate of 0.57%. During the 2019 epidemic, the age‐adjusted incidence had tripled to 6.27 cases per 1000 persons per year.^2^ However, neither neonatal infections nor vertical infections during pregnancy were reported in either the national surveillance data or during epidemics. We are reporting a rare case of serologically‐confirmed congenital dengue in a neonate with thrombocytopenia and ascites.

### 1.1 Case history and examination

Born to a primigravida with a 3‐day unspecified fever, a full‐term male neonate was delivered by emergent cesarean section due to presumed fetal distress at 37 4/7 weeks of gestation. The amniotic fluid was clear. He required positive pressure ventilation, with Apgar score of 7, 8 and 9 at 1, 5 and 10 min, respectively. He had normal anthropometric measurement: birth weight of 2750 gram (33rd percentile), head circumference 34 cm (65th), and length 49 cm (55th).

During pregnancy, the mother had received antenatal care regularly and had no pregnancy‐related complications. She had 3‐day history of fever and flu‐like symptoms prior to delivery. Her blood tests at admission (the delivery day) showed an increased C‐reactive protein (CRP) (72.4 mg/L) while leucocytes count (4.82 × 10^9^/L) and platelets (176 × 10^9^/L) were within normal range. Urine examination, SARS‐CoV‐2 Rapid Antigen Test, and hemoculture were warranted and showed negativity. Because of a spike in fever, Dengue NS1 Ag was done and showed positivity 4 h after the delivery of the baby. On the following day, it is worth noting that the mother developed thrombocytopenia (141 × 10^9^/L), and the lowest was 30 × 10^9^/L at Day 5 post‐partum, corresponding to Day 7 of the maternal dengue.

## 2 METHODS (DIFFERENTIAL DIAGNOSIS, INVESTIGATIONS AND TREATMENT)

**FIGURE 1:**
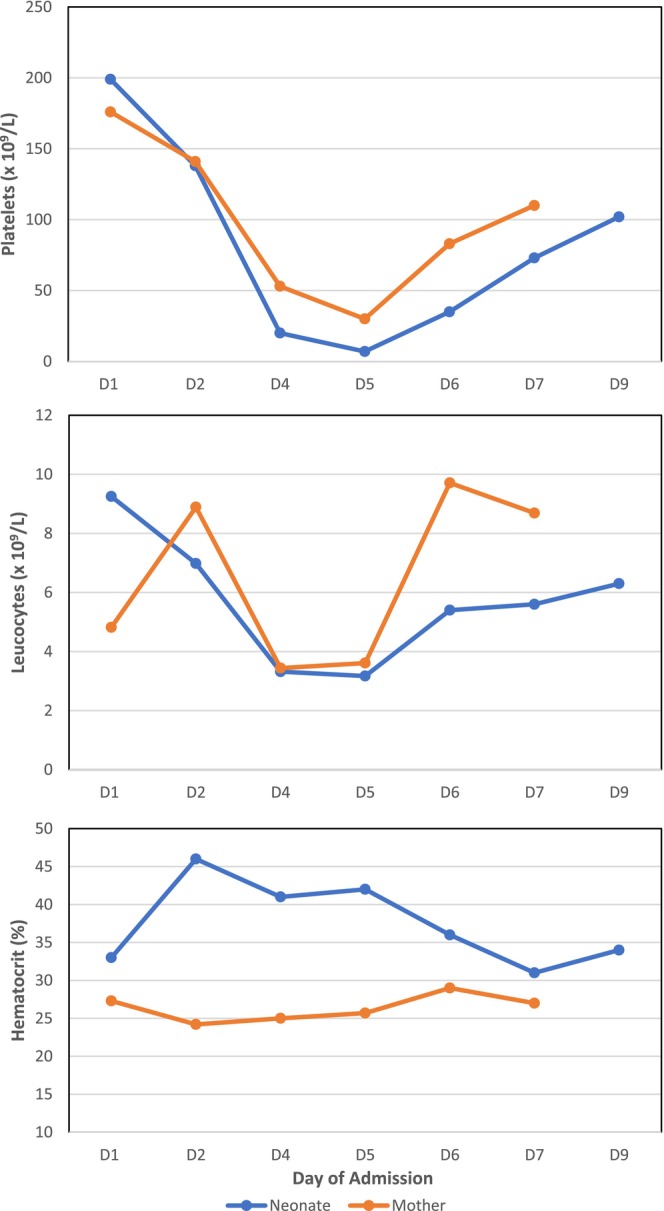
Hemogram of the neonate and mother during hospitalization.

The neonate was admitted to the Neonatal Intensive Care Unit (NICU) for respiratory distress and suspected early‐onset sepsis (EOS) in an early‐term neonate. Given the Silverman score of 3, he was put under Infant Flow CPAP for 12 h. Chest X‐ray was in favor of the transient tachypnea of newborn (TTN). In Figure 1, we demonstrated the hemogram of the mother and the baby from immediately after the delivery to Day 9 of life. Due to maternal fever and increased CRP, newborn's sepsis workup was done and revealed: normal leucocyte count (9.25 × 10^9^/L), with absolute neutrophil count of 6.29 × 10^9^/L, and Immature to Total neutrophil ratio (I/T ratio) of 0.03; hemoglobin and platelets were within normal range. Procalcitonin (PCT), however, was elevated to 100 mcg/L. Additional tests, including blood culture, gastric aspirate, syphilis serologic tests, and lumbar puncture, were warranted and showed negativity. Based on the local guidelines, empiric antibiotics (cefotaxime and ampicillin) were given. Because the maternal dengue NS1 Ag was positive 4 h after the delivery, dengue serology was conducted for the neonate, and the results came back negative (Table 1).

**TABLE 1:** Dengue serology of the neonate.

Day of hospitalization	D1	D2	D4	D5	D6
NS1 Ag	−	−		+	+
Dengue IgM	−	−		−	+
Dengue IgG	−	−		−	+
PCR			DENV positive		

At 48 h of life (D2), the neonate was weaned off oxygen and bottle‐fed well. However, he had an acute onset of febrile episodes (37.8°C–38.5°C), without skin rash or organomegaly. Physical examination was unremarkable: no bulging fontanel nor neurological signs. Blood tests were performed and showed mild thrombocytopenia (138 × 10^9^/L) and slightly increased CRP (12 mg/L). Dengue serology was controlled and showed negative results again.

**FIGURE 2:**
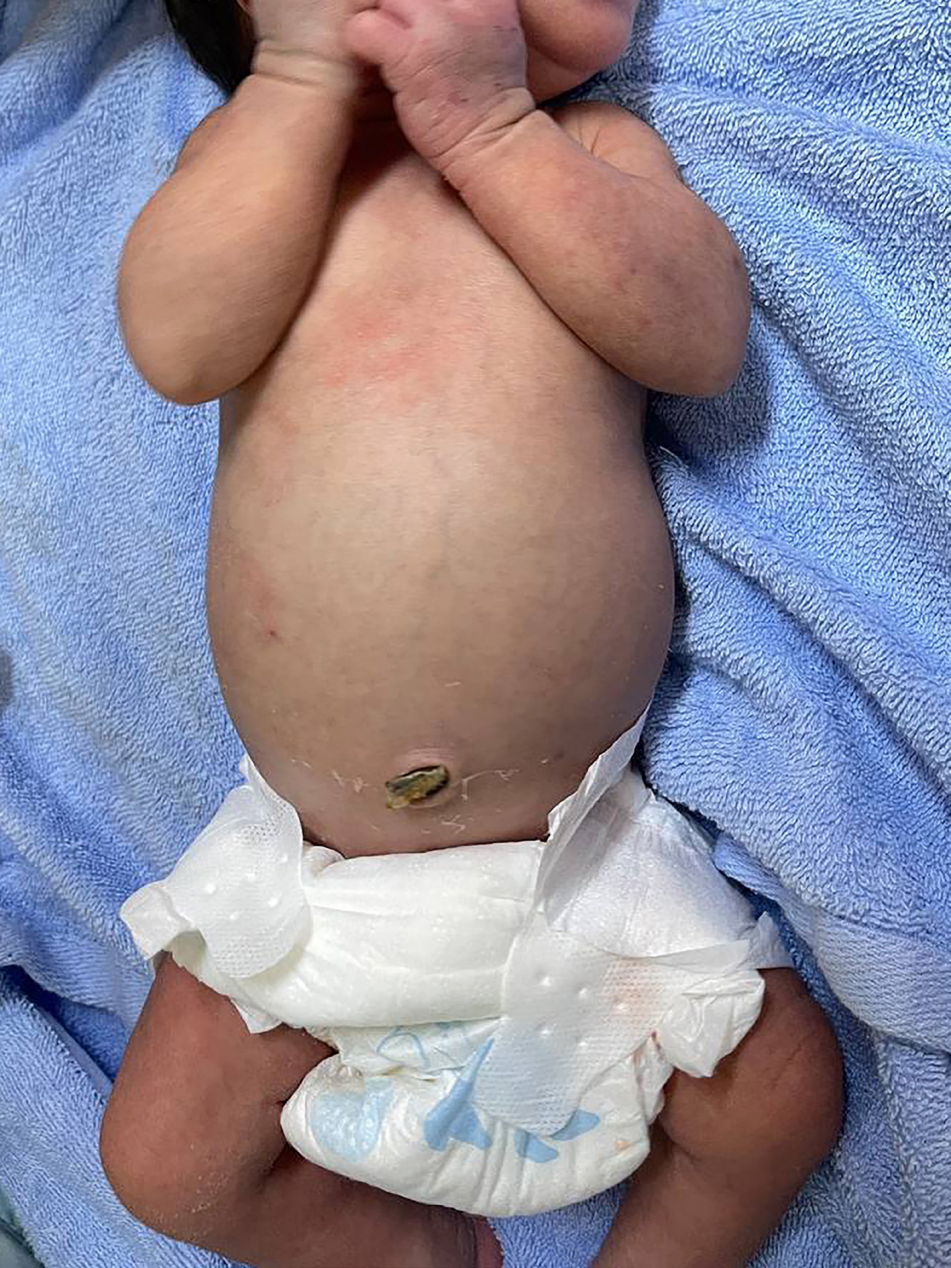
Abdominal distension on Day 3 due to ascites.

On day 3 (D3), he developed abdominal distension despite well‐tolerated feeding, associated with a soft and painless abdomen (Figure 2). Hemodynamics was stable and no episode of apnea was recorded. Plain abdominal radiography showed no signs of necrotizing enterocolitis (NEC), except for a diffusely increased density in the abdomen, which was later confirmed by bedside ultrasound to be moderate ascites. After revisiting the maternal medical report, particularly with severe and progressive neonatal thrombocytopenia and clinical fluid collection, Multiplex RT‐PCR was done and confirmed the diagnosis of congenital dengue on day 4 of life (Table 2).

**TABLE 2:** Virology report of the neonate.

Combined detection of Dengue/Zika/Chikungunya		
Method	Multiplex real‐time RT‐PCR	
Results	Zika	Not detected
	Dengue	Detected
	Chikungunya	Not detected
Conclusion	Infection with Dengue virus confirmed.	

### 2.1 Outcome and follow‐up

Fever subsided after 2 days (D4). Since he was active, with good feeding tolerance, the management was supportive with close observation of vital signs. Antibiotics were administered for 7 days and discontinued following the negative result of the second hemoculture. There were no bleeding signs. Neurological examination and brain ultrasound were normal. He received one pack of platelets due to severe thrombocytopenia (7 × 10^9^/L) at D5. Given clinical stability and progressively increased platelets, he was discharged at D12. At the follow‐up (D25), the boy was thriving well with normal physical examination and normal platelets.

## 3 DISCUSSION

Maternal consequences resulting from acquiring dengue fever during pregnancy are still debatable, which can be due to the heterogeneous findings across studies. Despite the controversy, the possible pregnancy‐related complications should not be overlooked. One 14‐year retrospective study conducted in French Guiana showed an association between dengue and an increased rate of various adverse outcomes in pregnancy including premature labor, premature birth, intrapartum hemorrhage, fetal demise, miscarriage, and retroplacental hematoma.^3^ Additionally, a study conducted during the outbreak in Ouagadougou reported that dengue‐infected expectant mothers faced twice the risk of adverse outcomes, including premature delivery and maternal death.^4^ Pregnant women are also identified as a high‐risk category and should be, according to WHO recommendations, transferred to and treated in hospitals where a multidisciplinary approach (obstetrics, medicine, and pediatrics) is available.^5^ However, unlike the Zika virus, dengue fever acquired during pregnancy has not been associated with any congenital defects.^6^, ^7^

Regarding the transmissibility of Dengue virus (DENV), it can be transmitted vertically from mothers to fetuses mostly during the third trimester, yet the rate of transmission is variable across different studies. The mother‐to‐child transmission was first recorded during the dengue epidemic in Tahiti in 1989, during which five neonates had IgM positive.^8^ The retrospective study (1992–2006) in French overseas Guiana found 3 positive cases out of 20 newborns' blood samples, corresponding to a rate of vertical transmission of 15%.^3^ In a large prospective study of 2531 Malaysian pregnant women in labor, one neonate out of 63 paired serology testing (maternal and umbilical cord) was positive, corresponding to a transmission rate of only 1.6%.^9^ However, a recent PCR‐based study during the outbreak in New Caledonia reported a vertical transmission rate of 90%. During the study period of 11 months, 9 out of 10 neonates, born to seropositive mothers who were infected and symptomatic 7 days prior and 2 days after the delivery, were positive.^10^

Clinical manifestations of dengue fever in neonates are variable and non‐specific in that the symptoms may resemble those of early‐onset neonatal sepsis. Although fever was the most common finding, ascites as a sign of plasma leakage were rarely documented.^10^, ^11^, ^12^, ^13^, ^14^, ^15^, ^16^, ^17^ According to the case series involving 17 neonates with congenital dengue, fever, and thrombocytopenia were reported in all infected neonates. In regard to the clinical progress, the fever typically occurred between 1 and 11 days, with a mean onset of 4 days and a mean duration of 3 days. The thrombocytopenia was observed shortly after the onset of fever, usually on day 6, with the average nadir of 19 × 10^9^/L.^11^ A more recent study including 9 vertically‐infected newborns underscored fever as a quasi‐constant symptom occurring in 8 cases (89%). Thrombocytopenia was recorded in 7 cases (78%), with platelet counts reaching a nadir between 20 and 83 × 10^9^/L.^10^

Diagnosis and management are also challenging in neonates. Dengue serologic diagnosis relies on the IgM that is detectable only after day 4 of the onset of fever.^18^ Reverse‐transcriptase polymerase chain reaction (RT‐PCR) and non‐structural protein 1 (NS1) are the confirmatory tests recommended by the Centers for Disease Control and Prevention (CDC) to diagnose the infection during the first week of illness.^19^ In terms of therapeutic interventions, unlike pediatric and adult populations, there are no specific guidelines for congenital dengue in neonates. The cornerstone of the treatment relies on symptomatic and supportive care in tandem with close observation.^17^

In our case study, the neonate, born to the mother with a three‐day fever prior to the delivery, was admitted for respiratory distress and suspected perinatal infection. Due to the increase of procalcitonin at birth and the local burden of bacterial sepsis, empiric antibiotics were prescribed. Additionally, syphilis tests were done because of the local resurgence of congenital syphilis.^20^, ^21^ Possible vertical transmission of DENV was suspected, yet two dengue serology tests were done after the post‐partum diagnosis of maternal dengue and after the onset of fever on the second day of life, both of which showed negativity. RT‐PCR assay was sought following the ultrasound diagnosis of ascites and serials of negative dengue NS1 antigen. Subsequently, given our recent report of Chikungunya neonatal infection,^22^ Multiplex PCR was done and confirmed the diagnosis of congenital dengue on day 4 of life. The management in our case was symptomatic and supportive, under close observation.

In conclusion, mother‐to‐child transmission of DENV should not be underestimated. Congenital dengue should be suspected in neonates with early‐onset sepsis, whose mothers present with intrapartum fever and come from an endemic region. The diagnosis can be challenging in the early days of life because clinical features are not specific and routine serology tests may not be informative. PCR remains the cornerstone to confirm the diagnosis of mosquito‐borne diseases.

## AUTHOR CONTRIBUTIONS

**Sakviseth Bin:** Conceptualization; data curation; investigation; methodology; writing – original draft; writing – review and editing. **Sethikar Im:** Supervision; writing – review and editing.

## FUNDING INFORMATION

There was no funding support for the case report.

## CONFLICT OF INTEREST STATEMENT

The authors declare no conflicts of interest.

## ETHICS STATEMENT

No ethical approval is required.

## CONSENT

Written informed consent was obtained from the patient's parents and is available for review upon request.

## Data Availability

The data that support the findings of this study are available from the corresponding author upon reasonable request.

## References

[1] Dengue and severe dengue . Geneva: World Health Organization. 2023 Accessed October 19 2023. https://www.who.int/news‐room/fact‐sheets/detail/dengue‐and‐severe‐dengue

[2] Yek C , Li Y , Pacheco AR , et al. National dengue surveillance, Cambodia 2002–2020. Bull World Health Organ. 2023;101(9):605‐616. 37638355 10.2471/BLT.23.289713 PMC10452936

[3] Basurko C , Carles G , Youssef M , Guindi WE . Maternal and foetal consequences of dengue fever during pregnancy. Eur J Obstet Gynecol Reprod Biol. 2009;147(1):29‐32. 19632027 10.1016/j.ejogrb.2009.06.028

[4] Tougma SA , Zoungrana/Yaméogo WN , Dahourou DL , et al. Dengue virus infection and pregnancy outcomes during the 2017 outbreak in Ouagadougou, Burkina Faso: a retrospective cohort study. PLoS One. 2020;15(9):e0238431. 32886677 10.1371/journal.pone.0238431 PMC7473539

[5] World Health Organization . Comprehensive Guideline for Prevention and Control of dengue and dengue Haemorrhagic Fever. 2011.

[6] Phongsamart W , Yoksan S , Vanaprapa N , Chokephaibulkit K . Dengue virus infection in late pregnancy and transmission to the infants. Pediatr Infect Dis J. 2008;27(6):500‐504. 18434933 10.1097/INF.0b013e318167917a

[7] Machain‐Williams C , Raga E , Baak‐Baak CM , Kiem S , Blitvich BJ , Ramos C . Maternal, fetal, and neonatal outcomes in pregnant dengue patients in Mexico. Biomed Res Int. 2018;2018:9643083. 29607328 10.1155/2018/9643083 PMC5828467

[8] Poli L , Chungue E , Soulignac O , et al. Apropos of 5 cases observed during the epidemic in Tahiti (1989). Bull Soc Pathol Exot. 1990;84(5 Pt 5):513‐521. 1819401

[9] Tan PC , Rajasingam G , Devi S , Omar SZ . Dengue infection in pregnancy: prevalence, vertical transmission, and pregnancy outcome. Obstet Gynecol. 2008;111(5):1111‐1117. 18448743 10.1097/AOG.0b013e31816a49fc

[10] Arragain L , Dupont‐Rouzeyrol M , O'Connor O , et al. Vertical transmission of dengue virus in the peripartum period and viral kinetics in newborns and breast milk: new data. J Pediatric Infect Dis Soc. 2017;6(4):324‐331. 27760799 10.1093/jpids/piw058

[11] Sirinavin S , Nuntnarumit P , Supapannachart S , Boonkasidecha S , Techasaensiri C , Yoksarn S . Vertical dengue infection: case reports and review. Pediatr Infect Dis J. 2004;23(11):1042‐1047. 15545860 10.1097/01.inf.0000143644.95692.0e

[12] Ranjan R , Kumar K , Nagar N . Congenital dengue infection: are we missing the diagnosis? Pediatr Infect Dis. 2016;8(4):120‐123.

[13] Chotigeat U , Kalayanarooj S , Nisalak A . Vertical transmission of dengue infection in Thai infants: two case reports. J Med Assoc Thai. 2003;86(Suppl 3):628‐632. 14700159

[14] Fatimil LE , Mollah AH , Ahmed S , Rahman M . Vertical transmission of dengue: first case report from Bangladesh. Southeast Asian J Trop Med Public Health. 2003;34(4):800‐803. 15115091

[15] Sinhabahu VP , Sathananthan R , Malavige GN . Perinatal transmission of dengue: a case report. BMC Res Notes. 2014;7:795. 25394748 10.1186/1756-0500-7-795 PMC4237779

[16] Aurpibul L , Khumlue P , Oberdorfer P . Dengue shock syndrome in an infant. BMJ Case Rep. 2014;2014:bcr2014205621. 10.1136/bcr-2014-205621 PMC4120039 25073530

[17] World Health Organization, & UNICEF . Handbook for Clinical Management of dengue. 2012.

[18] Simmons CP , Farrar JJ , van Vinh CN , Wills B . Dengue. N Engl J Med. 2012;366(15):1423‐1432. 22494122 10.1056/NEJMra1110265

[19] Centers for Disease Control and Prevention . Clinical Testing Guidance for Dengue. Accessed on May 18, 2024. https://www.cdc.gov/dengue/hcp/diagnosis‐testing/index.html 2024.

[20] Bin S , Im S . Congenital syphilis coinfection in a preterm infant with early onset sepsis due to Enterobacter cloacae. Case Rep Infect Dis. 2021;2021:1334846. 34336314 10.1155/2021/1334846 PMC8286191

[21] Bin S . Congenital pemphigus syphiliticus: a characteristic feature of a forgotten disease. BMJ Case Rep. 2021;14(10):e246310. 10.1136/bcr-2021-246310 PMC8499253 34620640

[22] Bin S , Phou K , Im S . Congenital chikungunya in a neonate with early‐onset sepsis and petechiae: an unusual case report. Clin Case Reports. 2023;11(1):e6896. 10.1002/ccr3.6896 PMC9871401 36703765

